# Potential functions of the shared bacterial taxa in the citrus leaf midribs determine the symptoms of Huanglongbing

**DOI:** 10.3389/fpls.2023.1270929

**Published:** 2023-11-14

**Authors:** Kaili Xia, Zengwei Feng, Xianjiao Zhang, Yang Zhou, Honghui Zhu, Qing Yao

**Affiliations:** ^1^ Key Laboratory of Biology and Genetic Improvement of Horticultural Crops (South China), Ministry of Agriculture and Rural Affairs, Guangdong Province Key Laboratory of Microbial Signals and Disease Control, College of Horticulture, South China Agricultural University, Guangzhou, China; ^2^ Key Laboratory of Agricultural Microbiomics and Precision Application (MARA), Guangdong Provincial Key Laboratory of Microbial Culture Collection and Application, Key Laboratory of Agricultural Microbiome (MARA), State Key Laboratory of Applied Microbiology Southern China, Institute of Microbiology, Guangdong Academy of Sciences, Guangzhou, China

**Keywords:** citrus, HLB -Huanglongbing, leaf midrib bacterial community, shared bacterial taxa, random forest, function prediction

## Abstract

**Instruction:**

Citrus is a globally important fruit tree whose microbiome plays a vital role in its growth, adaptability, and resistance to stress.

**Methods:**

With the high throughput sequencing of 16S rRNA genes, this study focused on analyzing the bacterial community, especially in the leaf midribs, of healthy and Huanglongbing (HLB)-infected plants.

**Results:**

We firstly identified the shared bacterial taxa in the midribs of both healthy and HLB-infected plants, and then analyzed their functions. Results showed that the shared bacterial taxa in midribs belonged to 62 genera, with approximately 1/3 of which modified in the infected samples. Furthermore, 366 metabolic pathways, 5851 proteins, and 1833 enzymes in the shared taxa were predicted. Among these, three metabolic pathways and one protein showed significant importance in HLB infection. With the random forest method, six genera were identified to be significantly important for HLB infection. Notably, four of these genera were also among the significantly different shared taxa. Further functional characterization of these four genera revealed that Pseudomonas and Erwinia likely contributed to plant defense against HLB, while Streptomyces might have implications for plant defense against HLB or the pathogenicity of Candidatus Liberibacter asiaticus (CLas).

**Disccusion:**

Overall, our study highlights that the functions of the shared taxa in leaf midribs are distinguished between healthy and HLB-infected plants, and these microbiome-based findings can contribute to the management and protection of citrus crops against CLas.

## 1 Introduction

Citrus is a crucial economic crop that faces numerous challenges, including diseases and pests such as Huanglongbing (HLB), citrus canker disease, and fruit flies. These issues have resulted in significant losses in citrus production worldwide (Rao et al., 2018; Zhou et al., 2021; Xiao et al., 2022). “Danxia Gonggan” (*Citrus reticulata Blanco* cv. Gonggan) is a geographical landmark agricultural product in Shaoguan City, Guangdong Province, China, that has also been affected by HLB in recent years (Munir et al., 2020). HLB is a quarantine disease that has been causing huge damage to citrus production in many regions. HLB is caused by the gram-negative bacterium *Candidatus* Liberibacter spp., and often referred to as citrus cancer due to the absence of known cure presently (Yang et al., 2021). The bacteria, including Asian (*C*Las, *Candidatus* Liberibacter asiaticus), African (*C*Laf), and American (*C*Lam) species, are confined to phloem sieve cells with *C*Las being the most widespread (Yang and Ancona, 2022). Current prevention and control measures are limited due to the difficulties in culturing the pathogen in pure cultures, and infected plants have to be removed to manage the disease (Pulici et al., 2022).

The plant microbiome, comprising microorganisms on and within various plant tissues (roots, leaves, seeds, etc.), plays a critical role in plant growth, nutrient absorption, tolerance to biotic and abiotic stresses, etc. (Turner et al., 2013; Vandenkoornhuyse et al., 2015; Li et al., 2022). For example, endophytic bacteria can enhance host plant growth and alleviate soil pollutants (Liu et al., 2022a), leaf microbiome shows significance in leaf water absorption (Rosado and Almeida, 2020), and soil microorganisms can enhance plant richness and productivity in grassland restoration (Abrahão et al., 2022). Further evidence has indicated that the rhizosphere microbiota can have varied impacts on disease resistance and nutrient uptake across different strawberry varieties, as reported by Lazcano et al. (2021). Pathogen infections, such as those caused by *Rhizoctonia solani* AG8 in barley, can alter the rhizosphere microbial community, leading to the accumulation of potentially antagonistic microorganisms (Yin et al., 2021). Additionally, certain bacterial isolates such as *Flavobacterium* TRM1 have been shown to inhibit bacterial wilt in susceptible tomato plants (Kwak et al., 2018). Collectively, these study highlight the crucial role of distinct microbiome in disease-resistant hosts.

The secondary metabolites produced by microorganisms in interaction with pathogenic bacteria in the host can enhance host resistance to these bacteria. For example, *Pseudomonas chlororaphis* subsp. *aurantiaca* strain zm-1 was found to potentially control peanut stem rot disease, because its phenazine secretion played a key role in disease prevention and thus resulted in a disease inhibition rate of up to 75.63% in pot experiments (Liu et al., 2022b). Similarly, the yeast species *Saccharomyces cerevisiae* and *Issatchenkia occidentalis* were observed to inhibit the virulence characteristics of *Candida albicans* via the production of phenethyl alcohol and tryptophol (Kunyeit et al., 2021). In another study, spraying citrus with secondary metabolites of *Pseudomonas aeruginosa* reduced bacterial titers in citrus leaves and upregulated host-related defense genes (Pistori et al., 2018). This study suggests that the secondary metabolites produced by the microbiome of resistant plants under stress conditions may be crucial for host resistance.

The functional groups present in plant microbiome have a significant impact on plant growth, environmental adaptability, and stress resistance (Podolich et al., 2015; Grady et al., 2019; White et al., 2019; Fadiji and Babalola, 2020; Simonin et al., 2020; Krabberød et al., 2022). On the other hand, a single plant has several ecological niches (e.g. rhizosphere, phyllosphere, endosphere) with varied physiological and biochemical features and thus different microbial community (Xiong et al., 2021). In many cases, microbes tend to show “home field advantage” effects in their functioning (Osburn et al., 2022; Jiang et al., 2023). A native synthetic bacterial community promoted plant growth of maize more greatly than a commercial plant growth-promoting rhizobacteria, because the native community colonized the soil better than the commercial agent and was more compatible with the resident community (Jiang et al., 2023). The citrus rhizosphere and leaf microbiota have been implicated in the occurrence and development of HLB (Wang et al., 2017; Ginnan et al., 2020), with strains isolated from citrus leaves possessing HLB resistance (Blacutt et al., 2020; Munir et al., 2022). These reports clearly indicate that native microbes might be more efficient in particular functionality than exotic microbes.

In this scenario, we hypothesized that endosphere bacterial community in leaf midribs is closely associated with HLB, considering that *C*Las occupies the unique ecological niche of phloem. However, there is no information available on the bacterial community in leaf midribs of healthy and infected citrus plants yet. Therefore, this study aimed to specifically analyze the bacterial community in the citrus leaf midribs of healthy and infected plants using high-throughput sequencing method. The shared taxa of healthy and infected leaf midribs were identified and characterized, and the antagonistic or risky species of *C*Las helper were recognized, which eventually contribute to the biological control of HLB.

## 2 Materials and methods

### 2.1 Sample collection

The leaf samples used in the study were collected from 10 citrus orchards located in 10 towns in Renhua County (24°56’ ~ 25°27’N, 113°30’ ~ 114°02’E) in Shaoguan City, Guangdong Province, China, which is one of the main production area of ‘Gonggan’ but suffered from HLB recently. From each orchard, 4~8 citrus plants with no HLB symptoms or HLB symptoms to different degree were selected randomly, and leaves distributing uniformly on the outside of canopy were sampled from each plant. The collected leaves were carefully placed in sterile bags. Finally, a total of 61 leaf samples were obtained for this study. All the samples were placed in an insulated ice box immediately after sampling and transported to the laboratory, where they were stored at a temperature of 4 °C until further processing.

### 2.2 Sample preparation

The leaf samples were sterilized using a rigorous protocol. Firstly, they were washed with 75% alcohol for 30 seconds in an ultra-clean workbench. Following this, the samples were washed with sterile water 3~5 times to eliminate any residual alcohol. Then, the samples underwent further sterilization with 2% sodium hypochlorite for 1 min and were subsequently washed again with sterile water 3~5 times. To ensure complete disinfection, the final elution solution from the cleaning process was collected. A 100 µL of this solution was plated on LB agar plates for microbial culturing. The absence of microbial growth after incubation confirmed successful complete disinfection. Next, the midrib (approximately 2 mm in width) was excised using a sterile surgical blade, and then cut into small pieces (approximately 5 mm in length) and placed in a sterile 2 mL centrifuge tube. The samples were ground into a fine powder using a homogenizer, and DNA extraction was performed using the Tiangen DP305-02 plant kit (Faddetta et al., 2021).

### 2.3 Molecular identification of Honglongbing

The quality of extracted DNA was assessed using a Nanodrop spectrophotometer and gel electrophoresis. For the PCR amplification of the 16S rRNA gene specific to *C*Las, the OI1/OI2c primers were used (Jagoueix et al., 1996). The PCR reaction was set up as follows: a 25 µL system containing 9.5 µL ddH_2_O, 12.5 µL 2×Taq PCR Master Mix, 1 µL 10 μM forward primer, 1 µL 10 μM reverse primer, and 1 µL DNA template. The PCR program consisted of an initial denaturation step at 95 °C for 3 min, followed by 34 cycles of denaturation at 95 °C for 30 s, annealing at 64 °C for 35 s, extension at 72 °C for 80 s, and a final extension step at 72 °C for 7 min. For the quantitative detection of *C*Las using qPCR, specific primers *C*Las-4G and HLBr were used, along with the HLBp probe (Bao et al., 2020). The qPCR reaction was set up in a 20 µL system, including 10 µL SuperMix, 0.4 µL 10 μM forward primer, 0.4 µL 10 μM reverse primer, 0.4 µL 10 μM probe, 0.4 µL Passive Dye, 1 µL DNA template, and 7.4 µL nuclease-free water. The qPCR program included an initial incubation step at 50 °C for 2 min, followed by denaturation at 95 °C for 10 min, and 40 cycles of denaturation at 95 °C for 15 s and annealing/extension at 60 °C for 1 min.

The detection methods commonly used for HLB include PCR and qPCR, which are well-established for their accuracy and widespread use in HLB detection (Ángel et al., 2014). However, there is currently no consensus on the threshold for distinguishing between HLB-positive and -negative samples. Various studies have reported different threshold values, such as CT values of 32 (Mira et al., 2019), 33 (Paula et al., 2018), 36 (Turechek et al., 2012), or 40 (Bai et al., 2013). In this experiment, based on the PCR and qPCR results, a threshold for defining a healthy strain was set at a CT value greater than 34, while a CT value less than 34 was considered indicative of an HLB-positive strain. With this criteria, all the 61 midrib samples were divided into infected group (36 samples) and healthy group (25 samples) (**Supplementary Table 1**).

### 2.4 Amplicon sequencing and bioinformatics analysis

The 61 DNA samples were sent to Novogene for amplicon sequencing of the 16S rRNA gene. The sequencing was performed using the Illumina NovaSeq platform with a Paired_End approach. The V3~V4 region (341F: CCTAYGGGRBGCASCAG; 806R: GGACTACNNGGGTATCTAA T) was targeted for sequencing (SRR25558690-SRR25558750, https://submit.ncbi.nlm.nih.gov/subs/). After obtaining the raw data, a series of data processing steps were carried out. The barcode and primers were removed, followed by merging, filtering, and decontamination of the paired-end sequences using QIIME2. Taxonomic classification was performed using the SILVA database with a similarity threshold of 97% (Almeida et al., 2018). The resulting high-quality data was then analyzed using R version 4.0.2, utilizing packages such as ‘barplot’ and ‘vegan’ for creating stacked histograms. TBtools was used for constructing Venn diagrams (Chen et al., 2020), and ImageGP was used for producing PCoA diagrams. Function prediction was done using the ‘PICRUSt2’ package in R (Douglas et al., 2020), and importance ranking was determined using random forest analysis with the ‘RandomForest’ package in R (Shibahara et al., 2017), which mainly contains a random forest classification and selection of important variables contributing to the difference between groups. After removing low-quality and chimeric sequences, a total of 4,138,080 sequences were obtained for the midrib bacterial community. These sequences were further decontaminated to eliminate any host contamination. The data saturation was evaluated using a dilution curve, as shown in **Supplementary Figure 1**. Based on the curve, it can be concluded that the decontaminated data was suitable for subsequent analysis.

## 3 Results

### 3.1 Composition and diversity of bacterial communities in leaf midribs of healthy and infected plants

**Figure 1:**
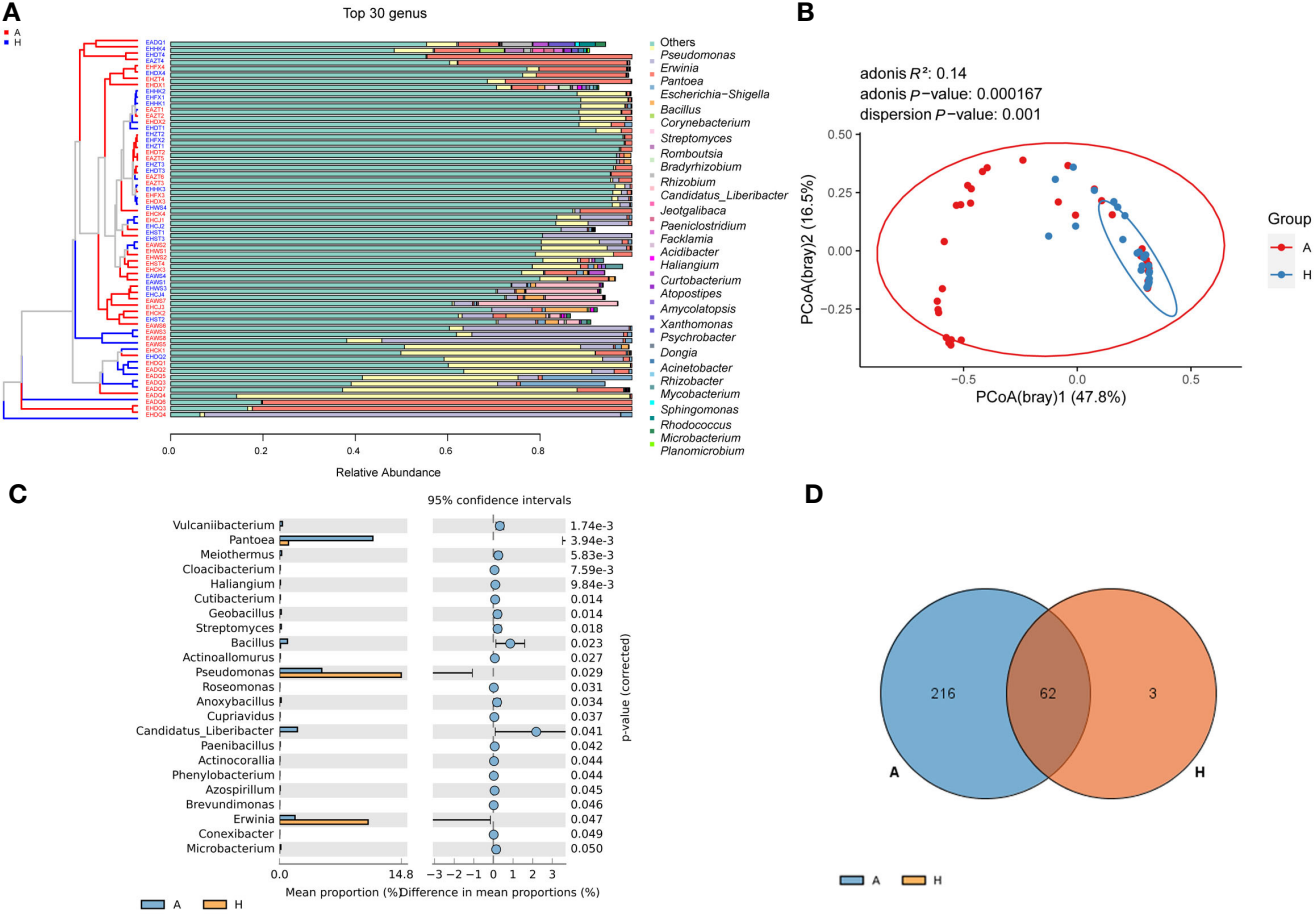
The composition of the bacterial community in the leaf midribs of healthy and HLB-infected citrus plants. **(A)** The bacterial community composition at the genus level with the top 30 genera shown. **(B)** PCoA analysis demonstrating the significant difference in the bacterial community structure at the genus level between two groups. **(C)** Bacterial taxa with significant differences in abundance between two groups. **(D)** Venn diagram showing the overlap and unique bacterial genera present in the midribs of two groups. A: infected group; H: healthy group.

After removing contamination and other undesired sequences, sequencing data was obtained from midrib samples, which revealed a total of 281 genera at the genus level. The top 30 genera accounted for 98.52% of the total abundance (**Figure 1A**; **Supplementary Table 2**). To analyze the community composition, we focused on these top 30 genera. Significant differences (*P* < 0.001) were observed in the bacterial community between healthy and infected plants (**Figure 1B**). Among the healthy plants, the most abundant genera were *Pseudomonas* (53.27%), *Erwinia* (37.36%), *Escherichia*-*Shigella* (4.90%), *Pantoea* (4.05%), and *Bacillus* (0.23%). In contrast, infected plants had the highest abundance of *Pantoea* (66.01%), followed by *Pseudomonas* (13.38%), *Erwinia* (7.06%), *Corynebacterium* (0.90%), *Streptomyces* (0.86%), *Romboutsia* (0.72%), and *Bacillus* (0.70%) (**Figure 1A**; **Supplementary Table 3**). Among the 281 genera, 23 genera showed significant differences in abundance between the two groups (**Figure 1C**). Additionally, the abundance of *Pseudomonas* and *Erwinia* significantly decreased in infected citrus compared to healthy ones. Conversely, the abundance of *Pantoea*, *Bacillus*, *Vulcaniibacterium*, *Meiothermus*, *Streptomyces*, pathogen *C*Las and etc. significantly increased in infected samples (**Figure 1C**). Furthermore, compared to healthy samples, the α diversity index showed a significant increase (Chao1), but a significant decrease (Good’s coverage) in infected samples. The overall diversity (Shannon and Simpson indices) also tended to decrease in infected samples (**Table 1**). These results suggest that the bacterial communities in the leaf midribs of citrus plants underwent significant changes due to HLB infection.

**Table 1:** Diversity index of bacterial communities in the leaf midribs of healthy and HLB-infected citrus plants.

Groups	Shannon	Simpson	Chao1	Goods_coverage
A	0.9912	0.4580	39.1115	1.4057
H	1.1762	0.6157	35.9199	2.1006
*P* values	0.24386	0.08443	0.0069**	0.00003***

A: infected group; H: healthy group. P values were calculated using t-test. ** and *** indicate significant difference between A and H groups at P = 0.01 and P = 0.001 level.

### 3.2 The composition and differences of shared taxa in leaf midribs of healthy and infected plants

In total, 62 genera were found to be present in both healthy and infected citrus midribs (**Figure 1D**; **Supplementary Table 4**), which were considered hereafter as shared taxa between healthy and infected leaf midribs. These shared taxa mainly included *Pseudomonas*, *Erwinia*, *Pantoea*, *Bacillus*, and others. Additionally, the infected group exhibited 216 unique genera, which was much higher in number than the healthy group (3 unique genera). This suggests an increased bacterial richness in infected midribs compared to healthy ones (**Figure 1D**; **Table 1**).

**Figure 2:**
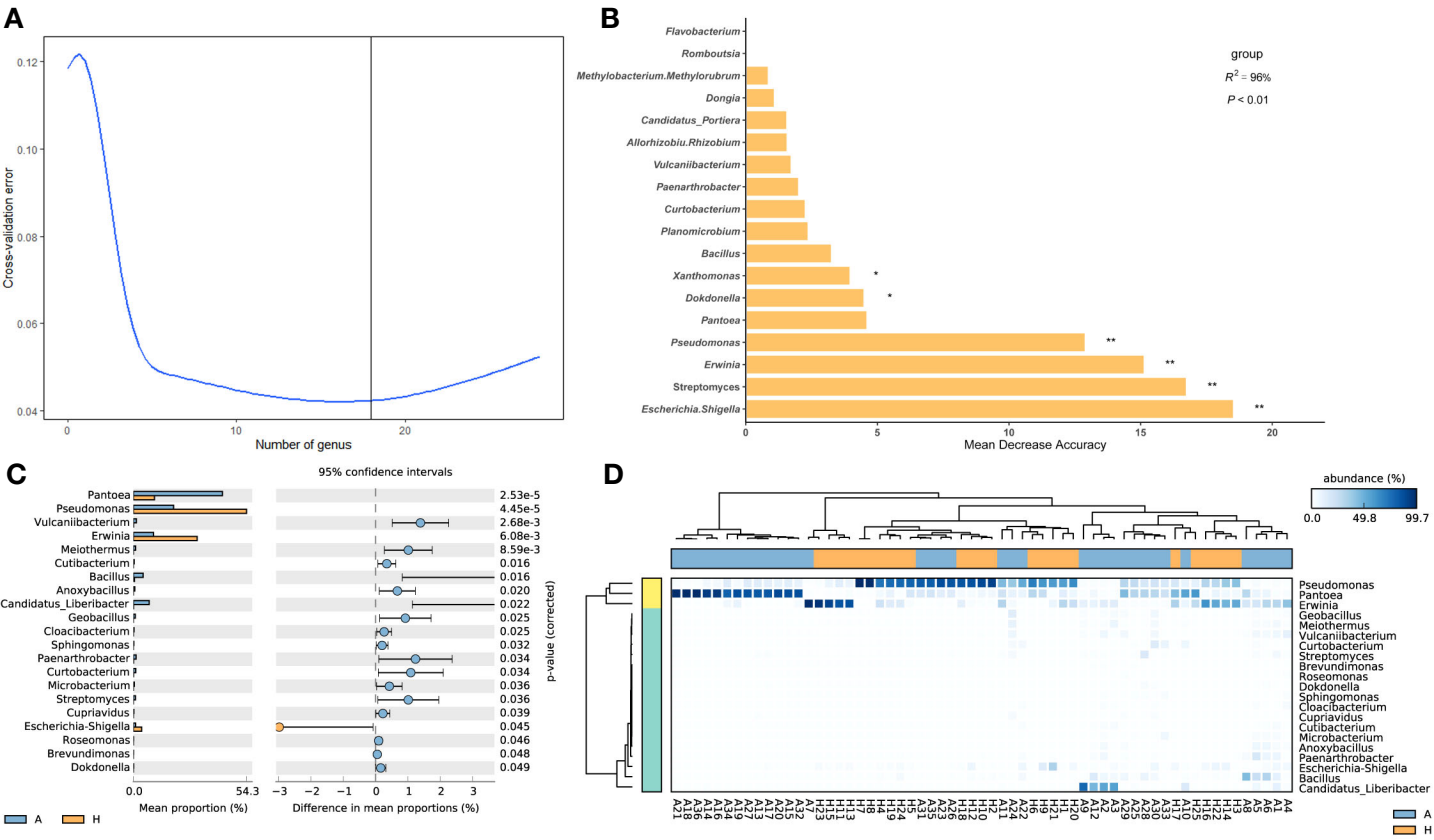
Importance ranking of and intergroup differences in shared bacterial taxa in the leaf midribs of healthy and HLB-infected citrus plants. **(A)** Cross validation (with random forest) results of shared taxa. **(B)** Random forest importance ranking of shared taxa in terms of their contribution to the intergroup difference. **(C)** The shared taxa with significant differences in abundance between two groups. **(D)** Heatmap showing the shared taxa with significant differences between two groups and their abundances. A: infected group, H: healthy group. * and ** indicate significant contribution of shared taxa to the intergroup difference at P = 0.05 and 0.01 level, respectively.

To analyze the differences in shared taxa between healthy and infected midribs and identify which taxa contribute to these differences, a random forest importance ranking was conducted using the shared bacterial community. The results showed that the model had the lowest error ratio when considering the top 18 genera (**Figure 2A**). The importance values, known as Mean Decrease Accuracy, were then used to rank the genera. The analysis revealed that 6 genera were of significant importance: *Escherichia*-*Shigella*, *Streptomyces*, *Erwinia*, *Pseudomonas*, *Xanthomonas*, and *Dokdonella* (**Figure 2B**). Among these, 4 genera (*Escherichia*-*Shigella*, *Streptomyces*, *Erwinia*, and *Pseudomonas*) showed significant differences between healthy and infected midribs. *Streptomyces* was found to be significantly enriched in infected midribs, while the other three genera were significantly enriched in the healthy samples (**Figure 2C**). This result suggests that these 4 genera were closely associated with the onset of HLB in citrus plants.

Additionally, among the 62 genera, 21 genera exhibited significant differences in abundance between healthy group and infected group (**Figure 2C**), indicating that approximately one-third of the shared taxa underwent changes in abundance in association with HLB. In detail, *Pseudomonas* and *Erwinia* were significantly enriched in healthy midribs, while *Pantoea*, *Bacillus*, and the pathogenic bacteria *Candidatus* liberibacter were significantly enriched in infected samples. These findings suggest that *Pantoea* and *Bacillus* might be considered as risky species or pathogen helper while *Pseudomonas* and *Erwinia* might play important roles in the growth and development of citrus plants, or even possess antagonistic properties against *C*Las. However, the healthy group and the infected group could not be well distinguished according to the shared taxa (**Figure 2D**).

### 3.3 Function prediction of shared taxa in leaf midribs of healthy and infected plants

#### 3.3.1 Metabolic pathways

**Figure 3:**
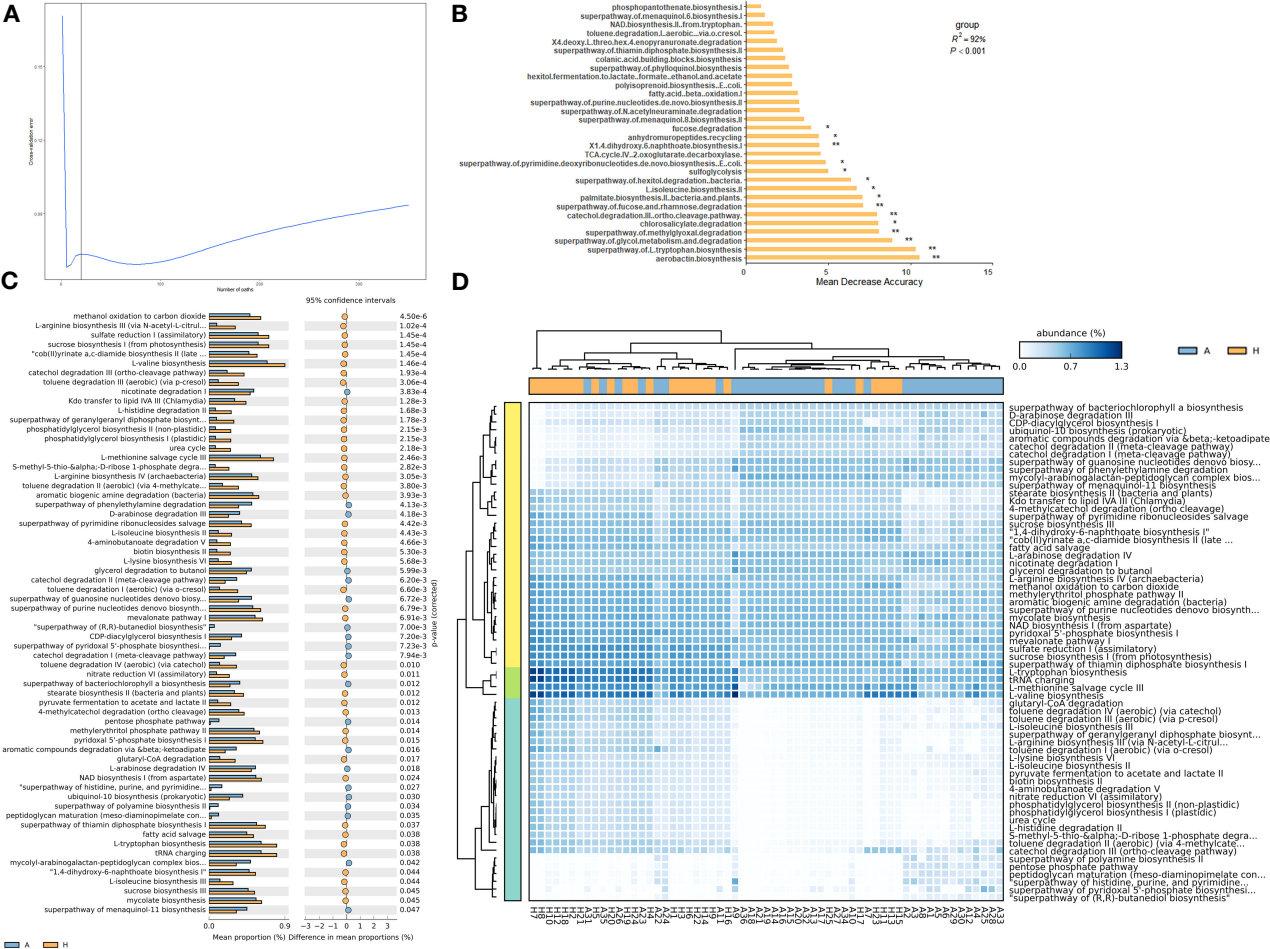
Importance ranking of and intergroup differences in the metabolic pathways (revealed by PICRUSt2) of shared bacterial taxa in the leaf midribs of healthy and HLB-infected citrus plants. **(A)** Cross validation (with random forest) results of the metabolic pathways of shared bacterial taxa. **(B)** Random forest importance ranking of the metabolic pathways of shared taxa in terms of their contribution to the intergroup difference. **(C)** The metabolic pathways with significant differences in abundance between two groups. **(D)** Heatmap showing the metabolic pathways with significant differences between two groups. A: infected group; H: healthy group. * and ** indicate significant contribution of shared taxa to the intergroup difference at P = 0.05 and 0.01 level, respectively.

To analyze the functional profiles of shared taxa in leaf midribs and identify intergroup differences, PICRUSt2 functional prediction was performed. Finally, 366 metabolic pathways (**Supplementary Table 5**), 5851 proteins (**Supplementary Table 6**), and 1833 enzymes (**Supplementary Table 7**) were predicted. To determine which pathways contributed to the shared taxa, a random forest importance ranking was conducted on the 366 metabolic pathways. The model had the lowest error ratio when approximately 30 metabolic pathways were in the fitting curve (**Figure 3A**), therefore we identified the top 30 pathways based on their importance value (Mean Decrease Accuracy). Among these, 15 pathways were significantly important (**Figure 3B**), including airborne biosynthesis, hyperpathway of L-tryptophan biosynthesis, chlorosalicylate degradation, and others.

Meanwhile, we identified 64 metabolic pathways significantly different in abundance between the bacterial communities in the healthy and infected midribs, including L-isoleucine biosynthesis II, catechol degradation III (ortho clearance path), and 4-aminobutanoate degradation V, and others. Among these, 44 pathways were significantly enriched in healthy samples, while 20 pathways were significantly enriched in infected samples (**Figure 3C**). Interestingly, 1,4-dihydroxy-6-naphthoate biosynthesis I, catechol degradation III ortho cleavage pathway, and L-isoleucine biosynthesis II were also found to be significantly important metabolic pathways (**Figure 3B**). More importantly, with these metabolic pathways with significant intergroup differences, we were able to distinguish between the healthy and infected midribs (**Figure 3D**), which suggests that the differences in metabolic pathways might contribute to the onset of HLB.

#### 3.3.2 Proteins

**Figure 4:**
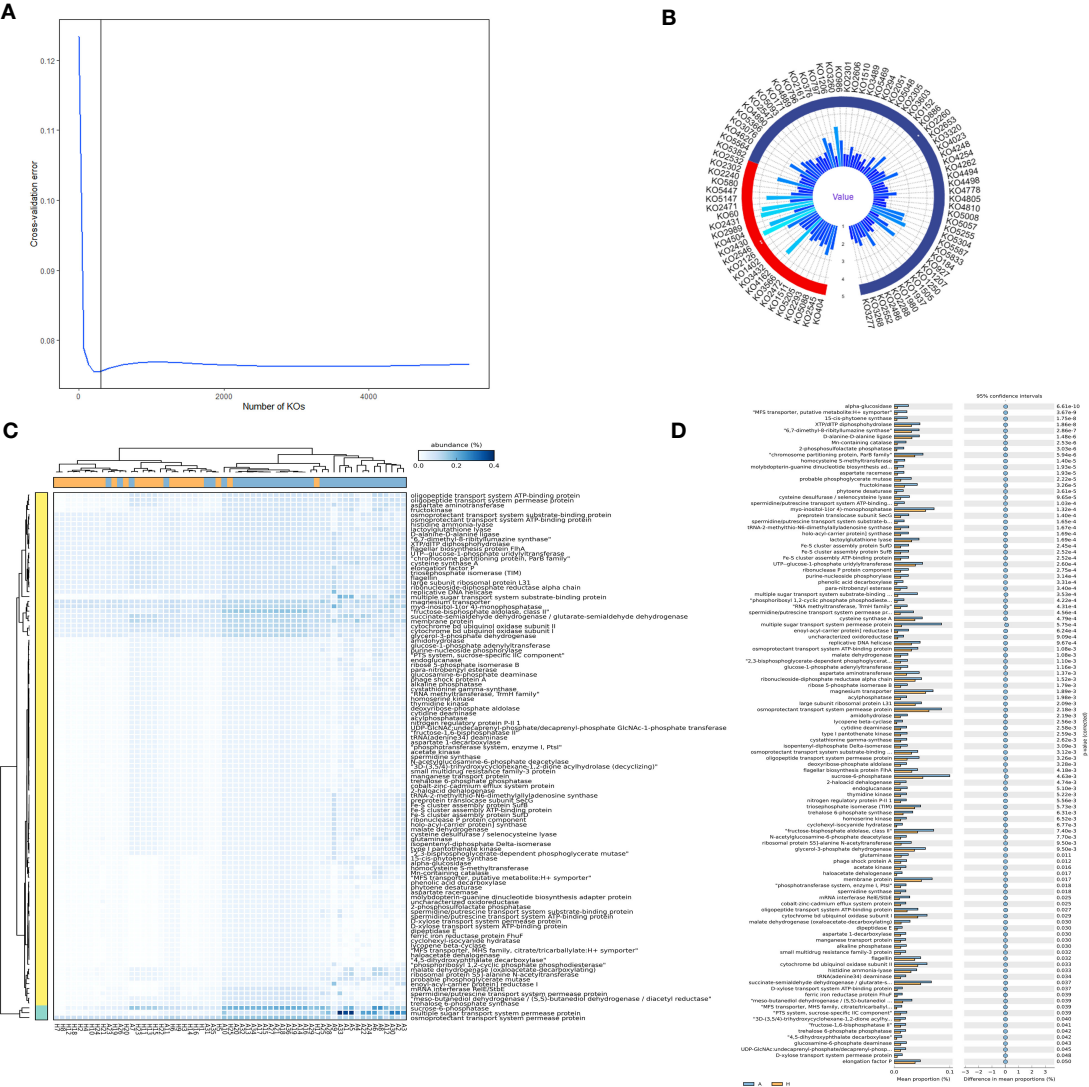
Importance ranking of and intergroup differences in the proteins (revealed by PICRUSt2) of shared bacterial taxa in the leaf midribs of healthy and HLB-infected citrus plants. **(A)** Cross validation (with random forest) results of the proteins of shared bacterial taxa. **(B)** Random forest importance ranking of the proteins of shared taxa in terms of their contribution to the intergroup difference. **(C)** The proteins with significant differences in abundance between two groups. **(D)** Heatmap showing the proteins with significant differences between two groups. A: infected group; H: healthy group.

To further ascertain the specific proteins responsible for the intergroup differences, a random forest importance ranking on 5,851 proteins was conducted. The data indicated that the error ratio reached the lowest point when approximately 300 proteins were included in the model (**Figure 4A**). Thus, we selected the top 300 proteins with the highest importance values (Mean Decrease Accuracy) for the importance ranking (**Figure 4B**). Finally, we identified 84 proteins that significantly or exceptionally contributed to shared taxa, including sn-glycerol 3-phosphate transport system ATP-binding protein, 3-methyl-crotonyl-CoA carboxylase alpha subunit, putative glycosyltransferase, and others, which likely played a crucial role in distinguishing between the healthy and infected groups (**Figure 4B**).

In addition, 109 proteins had significant intergroup differences in abundance, including 6,7-dimethyl-8-ribityllumazine synthase, fructose-1,6-bisphosphatase II, starch bisphosphate alpha, class II, and others (**Figure 4D**). Notably, all these significantly different proteins were enriched in the infected midribs, among which one particularly important protein was the spermidine/putrescine transport system ATP-binding protein. Furthermore, the heatmap of the significantly different proteins displayed distinct patterns, allowing for the differentiation of the two groups (**Figure 4C**). This indicates that the differences observed in the proteins of shared taxa were crucial in determining whether the plant is infected by *C*Las.

#### 3.3.3 Enzymes

**Figure 5:**
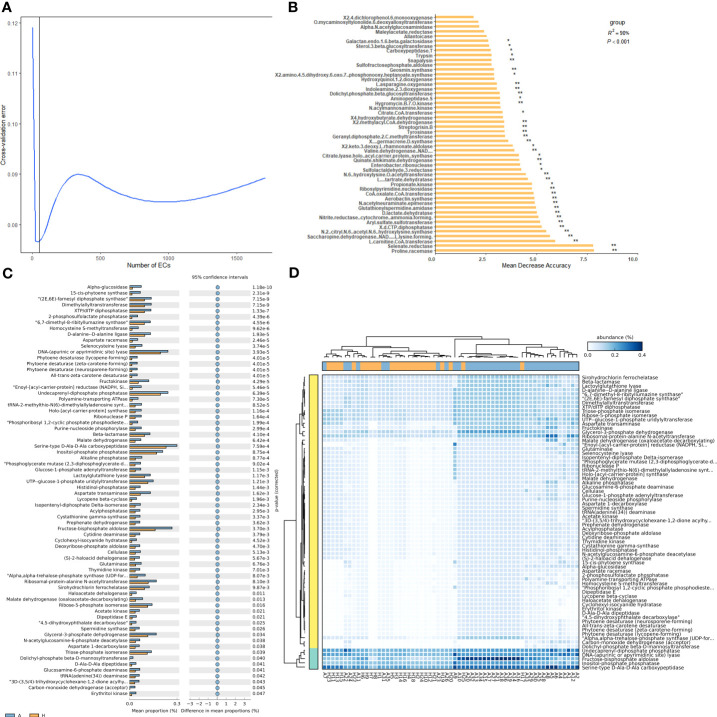
Importance ranking of and intergroup differences in the enzymes (revealed by PICRUSt2) of shared bacterial taxa in the leaf midribs of healthy and HLB-infected citrus plants. **(A)** Cross validation (with random forest) results of the enzymes of shared bacterial taxa. **(B)** Random forest importance ranking of the enzymes of shared taxa in terms of their contribution to the intergroup difference. **(C)** The enzymes with significant differences in abundance between two groups. **(D)** Heatmap showing the enzymes with significant differences between two groups. A: infected group; H: healthy group. * and ** indicate significant contribution of shared taxa to the intergroup difference at P = 0.05 and 0.01 level, respectively.

To determine the enzymes that contribute to the intergroup differences, we conducted random forest importance ranking on 1,833 enzymes. The results indicated that the lowest error ratio was achieved when the fitting curve included approximately 50 enzymes (**Figure 5A**). Based on the importance value (Mean Decrease Accuracy), we identified 41 enzymes with significant importance, including selenate reductase, 2-methylacyl CoA dehydrogenase, N2-citryl-N6-acetyl-N6-hydroxylysine synthase, and etc. (**Figure 5B**). These enzymes are likely important in distinguishing between healthy and infected groups.

Among 1833 enzymes, we further identified 70 enzymes with significant differences in abundance between the healthy and infected midribs. Notably, all these differential enzymes were enriched in the infected samples, including XTP/dITP diphosphatase, UTP-glucose-1-phosphate uridyltransferase, and Undecaprenyl diphosphate, and etc. (**Figure 5C**). Interestingly, none of these differential enzymes were identified to be important with the random forest analysis. In spite of this, with these differential enzymes, healthy and infected groups could be distinguished well (**Figure 5D**). Therefore, this analysis highlights the association of these differential enzymes with the onset of HLB.

### 3.4 Functional characterization of specific taxa

In order to better understand the functional implications of the differences in shared taxa between the healthy and infected groups, we focused on four genera (*Erwinia*, *Pseudomonas*, *Streptomyces*, *Scherichia-Shigella*) with significant contribution to the observed differences, and identified their proteins in association with disease resistance.

The results indicate that the enzyme glutathione S-transferase (K00799) was present in genera *Erwinia* (OTU3) and *Pseudomonas* (OTU4). This enzyme is known to be involved in detoxification processes and also plays a role in gibberellic acid (GA) biosynthesis. In the genus *Streptomyces* (OTU26), several proteins of interest were identified, including endo-1,4-β-xylanase (K01181), α-L-rhamnosidase (K05989), and 3-oxoacyl-[acyl carrier protein] reductase (K00059). Similar to the other genera, these proteins were enriched in the infected samples (**Table 2**; **Supplementary Tables 9–12**), suggesting that they might play a role in disease progression or the biochemical response of leaf midribs to infection by *C*Las.

**Table 2:** The proteins associated with potential disease resistance of shared taxa with significant importance and difference between healthy and HLB-infected groups.

Genus	Top OTU	KO proteins associated with disease resistance	Abundance	
			A	H
*Erwinia*	OTU3	K00799: Glutathione S-transferase	23717.185	17958.214
*Pseudomonas*	OTU4	K00799: Glutathione S-transferase	23717.185	17958.214
		K00059: 3-oxoacyl-[acyl-carrier protein] reductase	15880.822	11753.342
*Streptomyces*	OTU26	K01181: Endo-1,4-β-xylanase	198.975	79.192
		K05989: α-L-rhamnosidase	90.806	19.904
		K00059: 3-oxoacyl-[acyl carrier protein] reductase	15880.822	11753.342
*Escherichia*-*Shigella*	OTU450	None		

Top OTU indicates the most abundant OTU in the respective genus. A: infected group; H: healthy group.

Similarly, the gluconic acid 2-dehydrogenase (EC:1.1.99.3) were observed in *Erwinia*, while 3-oxoacyl-[acyl-carrier-protein] reductase (EC:1.1.1.100) was observed in *Pseudomonas*, in addition to glutathione S-transferase (EC:2.5.1.18). Furthermore, several enzymes, such as cellulases (EC:3.2.1.4), α-L-rhamnosidase (EC:3.2.1.40), endo-1,4-β-xylanase (EC:3.2.1.8), and 3-oxoacyl-[acyl carrier protein] reductase (EC:1.1.1.100) were observed in *Streptomyces*. Notably, all of these enzymes were found to be enriched in the diseased samples (**Table 3**; **Supplementary Tables S13–16**), suggesting their potential involvement in disease development or the response of infected midribs to pathogens.

**Table 3:** The enzymes associated with potential disease resistance of shared taxa with significant importance and difference between healthy and HLB-infected groups.

Genus	Top OTU	EC enzymes associated with disease resistance	Abundance	
			A	H
*Erwinia*	OTU3	EC 1.1.99.3: Gluconic acid 2-dehydrogenase	24553.033	19378.537
		EC 2.5.1.18: Glutathione S-transferase	23717.185	17958.214
*Pseudomonas*	OTU4	EC 1.1.1.100: 3-oxoacyl-[acyl-carrier-protein] reductase	15885.475	11753.822
		EC 2.5.1.18: Glutathione S-transferase	23717.185	17958.214
*Streptomyces*	OTU26	EC 3.2.1.4: Cellulases	4321.116	3303.067
		EC 3.2.1.40: α-L-rhamnosidase	90.806	19.904
		EC 3.2.1.8: Endo-1,4- β- Xylanase	198.975	79.1924
		EC 1.1.1.100: 3-oxoacyl-[acyl-carrier-protein] reductase	15885.475	11753.822
*Escherichia*-*Shigella*	OTU450	None		

Top OTU indicates the most abundant OTU in the respective genus. A: infected group; H: healthy group.

## 4 Discussion

### 4.1 Diversity of bacteria in leaf midribs of citrus plants

Our study demonstrates that HLB infection decreased the diversity but increased the richness of bacterial community in the leaf midribs of citrus plants. Previous studies revealed that the diversity and richness of bacteria in infected midribs increased, while specific key groups in leaves and roots decreased in the early stages of HLB infection, followed by an enrichment of beneficial taxa (Blaustein et al., 2017; Ginnan et al., 2020). Additionally, it has been found that when *Citrus reticulata* cv. Shatangju was invaded by *C*Las, the alpha diversity of its bacterial community initially decreased and then increased, but not reaching a significant level (Yan et al., 2021). Overall, the invasion of pathogenic bacteria is a dynamic process (Tang et al., 2020), so the diversity of plant microbiome may fluctuate depending on the period of invasion and sampling used.

### 4.2 Composition of shared taxa in healthy and infected midribs of citrus plants

In this study, the shared taxa in leaf midribs consisted of 62 genera, including *Pseudomonas*, *Erwinia*, *Pantoea*, and *Bacillus*, etc. These shared taxa have been commonly found in various crops such as barley, rice, sugarcane, grapes, soybeans, *Arabidopsis*, and in citrus plants as well. For instance, *Streptomyces*, *Sphingomonas*, *Kaitobacter*, and *Bacillus* have been identified as shared taxa in citrus rhizosphere by Penyalver et al. (2022). Similarly, *Pseudomonas*, *Sphingomonas*, and *Streptomyces* were identified as shared taxa in the rhizosphere, root endosphere, flower and flush of citrus in California by Xi et al. (2023). Additionally, according to a large number of literature, *Pseudomonas*, *Sphingobium*, *Chittinopaga*, and *Agrobacterium* were identified as shared taxa in citrus (see the review by Srivastava et al., 2022). These reports suggest that there was some variation in the composition of shared taxa among different citrus varieties, and this variation may be attributed to the variations in the surrounding environments (Trivedi et al., 2020) or the genetic makeup of the host. Although these reported shared taxa are derived from multiple niches other than leaf midribs, the bacteria can move between different niches (Xiong et al., 2021). Therefore, the shared taxa of a specific niche might be the shared taxa of another niche as well.

It is worth mentioning that, in this study, these shared taxa were found in both the healthy and the infected midribs. Therefore, it is likely that these taxa in the healthy midribs are involved in the plant growth and development while these taxa in the infected midribs are involved in disease resistance. For example, the shared taxa in maize plants are active nitrogen fixers or contribute to biological nitrogen fixation (Zhang et al., 2022), which demonstrates that the core taxa shared in all xylem sap samples were involved in plant growth and development. The shared core microbiota of soil microbiomes plays a crucial role in maintaining the functional stability of soil microbiota in afforestation ecosystems (Jiao et al., 2022). Additionally, the rotation between chili/eggplant and banana crops generates nine unique antagonistic shared core species, predominantly including *Bacillus* and *Pseudomonas*, which have significant impacts on the reduction in the incidence rate of banana wilt (Hong et al., 2023). These antagonistic shared core species persisted in high abundance in the rhizosphere soils of both chili/eggplant and banana in the rotation system compared to the banana monoculture system. Therefore, the shared taxa may play important roles in plant health, nitrogen fixation, ecosystem stability, and disease management. On the other hand, microbial functions highly depend on environments and a given microbial taxa can function differently in different environments (Djemiel et al., 2022). In this study, we identified the shared taxa in leaf midribs of citrus, but did not isolate them. It is necessary to perform the isolation and to test their potential functions in alleviating HLB in the future.

Our study identified 62 genera as shared taxa, accounting for 99.36% of all taxa in terms of abundance (infected group 98.27%, healthy group 99.94%). This suggests that the shared taxa were abundant in citrus midribs, with the unique taxa only accounting for 0.64% of all taxa (infected group 1.73%, healthy group 0.06%). Although the abundance of unique taxa was very low, they might play a crucial role in the health of citrus and thus deserve further investigation, especially considering that some of them were recruited by the infection by *C*Las. Interestingly, rare or key taxa can play a pivotal role in determining the structure and function of microbiomes (Banerjee et al., 2018). Rare taxa exhibit a wide range of environmental adaptation and have less functional redundancy compared to abundant taxa (He et al., 2022b), contribute significantly to the health and functioning of ecosystems (Lyons et al., 2005). Thus, it is crucial to consider the role of rare taxa in maintaining the overall health and functioning of the citrus microbiome. Further investigation on the specific functions and interactions of rare taxa with *C*Las might provide valuable insights into the mechanisms underlying HLB and the solutions to mitigate its impact.

### 4.3 Functional prediction of shared taxa in healthy and infected midribs of citrus plants

Our study identified three metabolic pathways with significant importance to the shared taxa, i.e., 1,4-dihydroxy 6-naphthoate biosynthesis I, categol degradation III ortho cleavage pathway, and L-isoleucine biosynthesis II. These pathways were were also enriched in healthy midribs. Interestingly, 1,4-dihydroxy 6-naphthoate, as the precursor of menaquinone, is related to the biosynthesis of menadione (MK), which is a potential target for evaluating antibiotics in Gram-positive bacteria (Hiratsuka et al., 2008; Choi et al., 2017). This suggests that the enrichment of 1,4-dihydroxy 6-naphthoate and other related metabolites in the healthy midribs could be attributed to antagonistic taxa contributing to antibacterial processes and maintaining plant health. Additionally, the categol degradation III ortho clearance pathway is involved in the degradation of aromatic hydrocarbon pollutants and environmental remediation (Heinaru et al., 2000; Basu et al., 2003; Murakami et al., 2003; Ontanon et al., 2015). L-methionine, another significantly important metabolic pathway, has antioxidant and free radical scavenging functions (He et al., 2022a). Meanwhile the effector protein encoded by *C*Las prophage has been reported to target ROS clearance-related proteins in citrus, inhibiting the accumulation of reactive oxygen species in plants and promoting the infection of pathogenic bacteria (Du et al., 2023). These information likely suggest that L-methionine may promote the invasion of *C*Las by clearing ROS.

Our comprehensive analysis of proteins revealed that the Spermidine/Putrescine transport system ATP-binding protein was not only a significantly important proteins, but also showed significant intergroup differences. Spermidine and putrescine are well recognized for their importance as polyamines in various cellular processes, including gene expression, cell growth, survival, stress response, and proliferation (Shah and Swiatlo, 2008; Gevrekci, 2017). Moreover, spermidine can promote the activity of the gene colibactin, and its intracellular pool in bacteria is tightly regulated through *de novo* synthesis and transport (Nanduri and Swiatlo, 2021). Therefore, maintaining polyamine homeostasis is crucial for proper bacterial physiology and has a significant impact on bacterial pathogenesis. Therefore, Yatin (2002) concludes that inhibitors of polyamine production shows promise in disease prevention and treatment. In these scenarios, it can be deduced that the Spermidine/Putrescine transport system ATP-binding protein identified in this study may play a vital role in the proliferation of *C*Las and the production of virulence factors. Further study is needed to understand the specific mechanisms involved.

In this study, it is clear that the metabolic pathways, proteins, and enzymes associated with significantly different shared taxa could distinguish between the healthy and infected groups, while the significantly different shared taxa could not. Therefore, it is reasonable to conclude that the functions (*i.e.* metabolic processes) but not the species of the shared taxa in midribs contributed to the onset of HLB. Microbes are normally metabolically versatile for better adaptation to changing environments, thus express only partial functional genes in a particular environment (Zhou et al., 2014; Koskella, 2020). Furthermore, the functionality of microbial community is more determined by environments than by its composition (Zhou et al., 2019). In summary, the functional differences in the significantly different shared taxa were instrumental in the symptoms of host HLB. Understanding these functional differences can provide insights into potential therapeutic targets and strategies for managing HLB.

### 4.4 Functional characterization of specific taxa

In this study, the functional characterization of the shared taxa revealed the presence of several proteins and enzymes significantly associated with the disease. One such protein was glutathione S-transferase, which acted as an antioxidant enzyme in oxidative stress and counteracted external stressors (Jin et al., 2022; Tang et al., 2023). The downregulation of SymE protein expression in infected citrus suggested its crucial role in restraining potential pathogens in healthy plants. Enzymes such as endo-1,4-β-xylanase and cellulases are well known for their ability to degrade cellulose and hemicellulose (Ariaeenejad et al., 2019; Thornbury et al., 2019; Thapa, 2020; Li et al., 2021), both of which are crucial components of plant cell walls. Therefore, the secretion of these enzymes by microorganisms is critical for their colonization inside the plant tissues and the interactions in host-pathogen-microbe relationships. The enzyme α-L-rhamnosidase efficiently hydrolyzed natural active substances like rutin (Bang et al., 2015; Yadav et al., 2017; Kalinova et al., 2018). Rutin, a flavonoid, functions in the growth and resistance of plants against both biotic and abiotic stress. Hence, α-L-rhamnosidase likely plays a crucial role in the citrus defense mechanisms against stress. Another enzyme, 3-oxoacyl-[acyl carrier protein] reductase, participates in the biosynthesis of fatty acids (Hu et al., 2018; Guo et al., 2019; Cross et al., 2021). Its overexpression can lead to an increase in the accumulation of fatty acids, thereby enhancing anti-oxidative activity and non-biotic stress resistance (Ye et al., 2022). Therefore, 3-oxoacyl-[acyl carrier protein] reductase may contribute to the defense against pathogenic bacteria in the citrus leaf midrib bacterial community. Gluconic acid 2-dehydrogenase is involved in the synthesis of gluconic acid, which can dissolve mineralized elements and promote their absorption in the host (de Werra et al., 2009; Yu et al., 2019; Jiménez-Gómez et al., 2020; Uroz et al., 2022). This may lead to an improvement of plant growth condition.

Thus, some bacterial taxa in the leaf midribs of citrus plants, such as *Erwinia*, *Pseudomonas*, and *etc.*, may engage in host defense against *C*Las, while *Streptomyces* may be involved in either the defense against *C*Las or the pathogenicity of *C*Las. Overall, the presence and activity of these specific proteins and enzymes in the midribs provide insights into the complexity of defense mechanisms and interactions between the host and its bacterial community in the context of disease. However, conflicting results have also been reported in other studies (Girard et al., 2020; Saldierna Guzmán et al., 2021; Yuan et al., 2021; Shen et al., 2022), which suggests that *Erwinia* and *Pseudomonas* may be opportunistic pathogens or isolates from *Erwinia* and *Pseudomonas* may be highly divergent in pathogenesis or beneficial nature (Girard et al., 2020). There are some isolates from these two genera being reported as plant growth promoting rhizobacteria (e.g., *Erwinia gerundensis*, *Pseudomonas fluorescens*) or pathogen (e.g., *Erwinia amylovora*, *Pseudomonas syringae*) so far.

## 5 Conclusion

**Figure 6:**
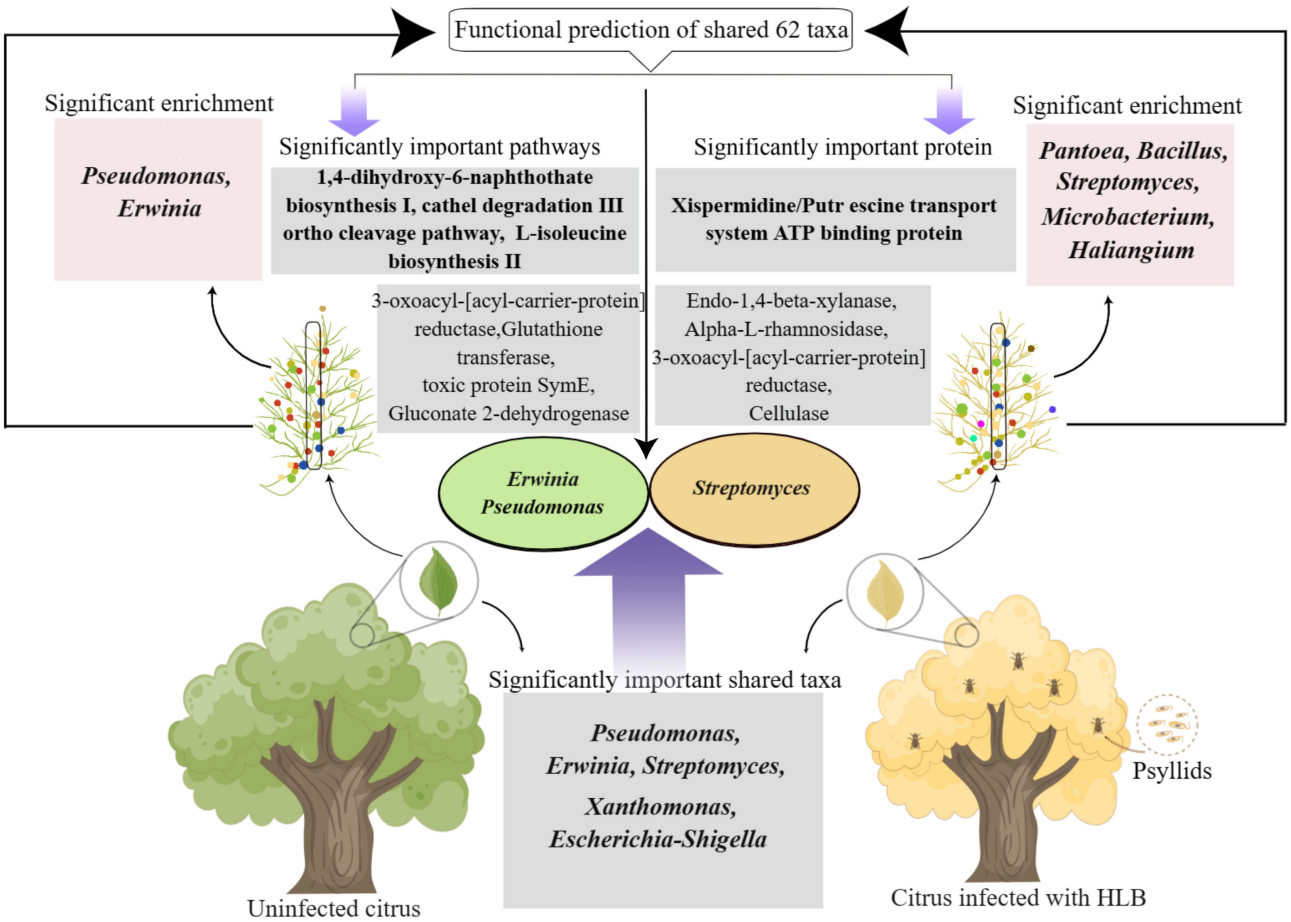
Diagram illustrating the involvement of shared bacterial taxa in the leaf midribs in the onset of citrus HLB.

*C*Las, the casual agent of HLB, inhabits phloem of citrus plants. To investigate the potential involvement of microbime in leaf midribs in the onset of HLB, we characterized the bacterial communities in midribs, and analyzed the relationship between their function and HLB (**Figure 6**). In summary, significant differences in bacterial communities the leaf midribs were observed between healthy and *C*Las infected citrus plants. Among the 62 genera shared between the two groups, 21 genera exhibited notable changes in abundance. Random forest analysis revealed that 6 genera significantly contributed to the differences between the groups, with 4 genera displaying significant differences in shared taxa. Further correlation analysis of functional data with these four genera indicated that *Erwinia* and *Pseudomonas* might participate in the defense against *C*Las, while *Streptomyces* might be involved in the defense against *C*Las or the pathogenic process of the *C*Las. Importantly, the result emphasized that the shared taxa with significant differences in pathways, proteins, and enzymes were able to distinguish between healthy and infected plants. Therefore, we conclude that the functional differences within the shared taxa in the leaf midribs of citrus plants determine the onset of HLB or not.

## Data availability statement

The datasets presented in this study can be found in online repositories. The names of the repository/repositories and accession number(s) can be found below: NCBI Sequence Read Archive, SRR25558690-SRR25558750.

## Author contributions

KX: Conceptualization, Formal analysis, Investigation, Methodology, Visualization, Writing – original draft. ZF: Formal analysis, Methodology, Writing – original draft. XZ: Investigation, Methodology, Resources, Writing – original draft. YZ: Methodology, Project administration, Resources, Writing – review & editing. HZ: Conceptualization, Funding acquisition, Supervision, Writing – review & editing. QY: Conceptualization, Funding acquisition, Supervision, Writing – review & editing.
