# Supplementary material for: Potential functions of the shared bacterial taxa in the citrus leaf midribs determine the symptoms of Huanglongbing
Source: Front Plant Sci. 2023 Nov 14;14:1270929. doi: 10.3389/fpls.2023.1270929 (PMC10682189; doi:10.3389/fpls.2023.1270929)
Supplement: Supplementary file 1 [file DataSheet_1.docx]

Supplementary Material

# Supplementary Figures and Tables

## Supplementary Figures


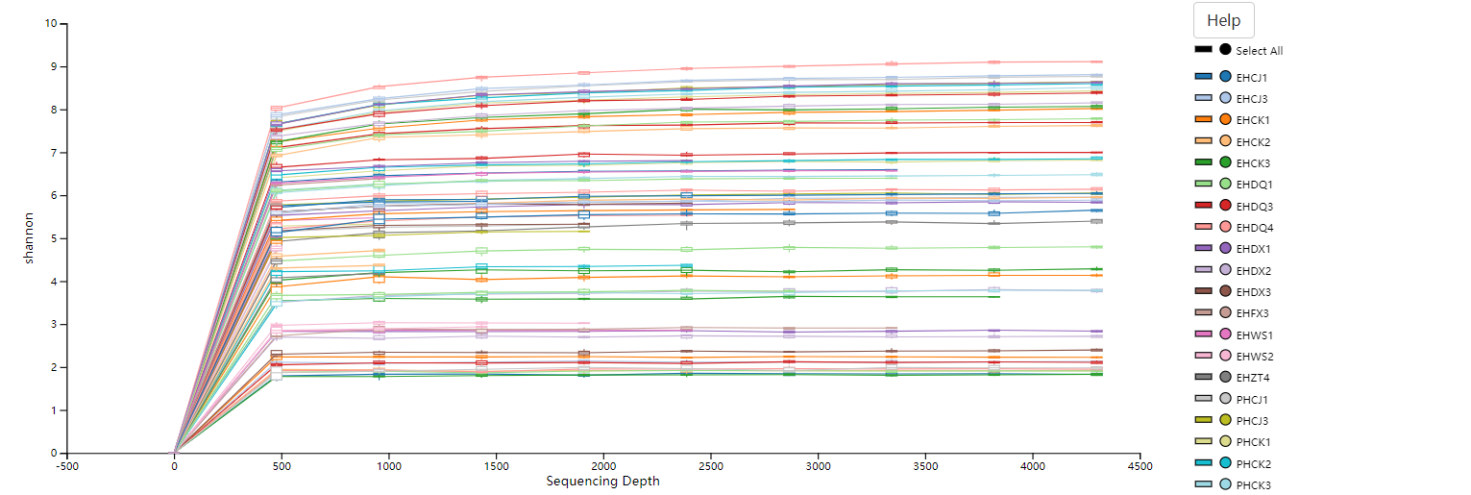


**Supplementary Figure 1.** Dilution curve of of citrus midribs microbial sequencing data after decontamination

## Supplementary Tables

**Supplementary Table 1.** qPCR test results of *C*Las and grouping in the citrus midribs. A: infected group; H: healthy group.

**Supplementary Table 2.** Relative abundance of the top 30 genera in bacterial communities in the citrus midribs.

**Supplementary Table 3.** Relative abundance of healthy and infected citrus midribs bacterial communities at the genus level.

**Supplementary Table 4.** Shared taxa of healthy and infected citrus midribs bacterial communities. A: infected group; H: Healthy group.

**Supplementary Table 5.** Prediction result of metabolic pathway in shared taxa.

**Supplementary Table 6.** Prediction result of proteins in shared taxa.

**Supplementary Table 7.** Prediction result of enzymes in shared taxa.

**Supplementary Table 8.** Results of random forest importance ranking with metabolic pathways.

**Supplementary Table 9.** Prediction result of proteins in *Erwinia*.

**Supplementary Table 10.** Prediction result of proteins in *Pseudomonas*.

**Supplementary Table 11.** Prediction result of proteins in *Streptomyces*.

**Supplementary Table 12.** Prediction result of proteinsin *Escherichia-Shigella*.

**Supplementary Table 13.** Prediction result of enzymes in *Erwinia*.

**Supplementary Table 14.** Prediction result of enzymes *Pseudomonas*.

**Supplementary Table 15.** Prediction result of enzymes *Streptomyces*.

**Supplementary Table 16.** Prediction result of enzymes *Escherichia-Shigella*.
